# Multivariate wind power curve modeling using multivariate adaptive regression splines and regression trees

**DOI:** 10.1371/journal.pone.0290316

**Published:** 2023-08-28

**Authors:** Khurram Mushtaq, Runmin Zou, Asim Waris, Kaifeng Yang, Ji Wang, Javaid Iqbal, Mohammed Jameel

**Affiliations:** 1 School of Automation, Central South University, Changsha, China; 2 School of Mechanical and Manufacturing Engineering, National University of Sciences & Technology, Islamabad, Pakistan; 3 School of Informatics, Communications and Media, University of Applied Sciences Upper Austria, Hagenberg, Austria; 4 Department of Civil Engineering, College of Engineering, King Khalid University, Asir, Abha, Saudi Arabia; UNITEN: Universiti Tenaga Nasional, MALAYSIA

## Abstract

Wind turbine power curve (WTPC) serves as an important tool for wind turbine condition monitoring and wind power forecasting. Due to complex environmental factors and technical issues of the wind turbines, there are many outliers and inconsistencies present in the recorded data, which cannot be removed through any pre-processing technique. However, the current WTPC models have limited ability to understand such complex relation between wind speed and wind power and have limited non-linear fitting ability, which limit their modelling accuracy. In this paper, the accuracy of the WTPC models is improved in two ways: first is by developing multivariate models and second is by proposing MARS as WTPC modeling technique. MARS is a regression-based flexible modeling technique that automatically models complex the nonlinearities in the data using spline functions. Experimental results show that by incorporating additional inputs the accuracy of the power curve estimation is significantly improved. Also by studying the error distribution it is proved that multivariate models successfully mitigate the adverse effect of hidden outliers, as their distribution has higher peaks and lesser standard deviation, which proves that the errors, are more converged to zero compared to the univariate models. Additionally, MARS with its superior non-linear fitting ability outperforms the compared methods in terms of the error metrics and ranks higher than regression trees and several other popular parametric and non-parametric methods. Finally, an outlier detection method is developed to remove the hidden outliers from the data using the error distribution of the modeled power curves.

## Introduction

As the world population increases, energy consumption is increasing by each day. However, to meet these demands, the use of fossil fuels causes severe damage to the environment, leading to global warming. To meet the challenge of decreasing the use of fossil fuel, the world is moving towards renewable resources for electrical power generation. Among many renewable energy technologies, wind energy generated through wind turbines is among the fastest-growing electricity generation source [1]. Wind energy is cheap, clean, unlimited, sustainable and extensively distributed [2]. Wind power produced more than 6 percent of global electricity in 2020 with 743 GW of global capacity as stated in the Global Wind Report 2021 [3].

In order to make wind energy competitive with non-renewable energy sources, there is a need to reduce the maintenance cost of the wind turbine. However, to reduce the maintenance cost, early fault detection should be made possible, because it speeds up the repair process, which reduces the non-operational time of the turbine and the resulting loss of energy. Hence, tracking the performance of a turbine plays a crucial role in early fault detection and helps in reducing the cost of mounting additional sensors on the turbine [4, 5]. Since wind energy is stochastic and non-linear in nature, hence to increase the use of wind power in the power grid, accurately predicting the power generated by wind turbines is necessary. Accurately predicting the power can help the power system to plan and manage accordingly and make energy management much easier. The wind turbine power curve (WTPC), which shows the non-linear relationship between wind speed and wind power [6], serves as an important tool for performance monitoring and obtaining accurate forecasts of future wind power [7]. A power curve model depicts the performance and behavior of a wind turbine in normal operating conditions. Hence, the performance of the wind turbine can be monitored by comparing the expected wind power on WTPC with the actual (measured) power, and anomalies and faults can be detected. WTPC can be used for wind power prediction if the corresponding forecasted wind speed value is available. Several methods are available for wind speed forecasting, such as the ones proposed by [8–10]. Furthermore, accurate WTPC models also help in wind energy potential estimation of the area and selection of a wind turbine [11].

Theoretical power curves which are given by wind turbine manufacturers based on the International Electrotechnical Commission (IEC) standard, cannot accurately depict the wind turbine performance because they are estimated under ideal environmental conditions [11, 12]. Wind turbines, on the other hand, are hardly used in ideal conditions, and real power curves may differ significantly from theoretical ones because they do not take into account complicated variables like the air density, wind field, wind direction, yaw, and pitch misalignment, shading effects from surrounding obstacles, mechanical and control issues, location of the turbine, as well as uncertainties in measurements [13, 14]. Hence, it is necessary to construct reliable WTPC models using data-driven approaches (which use wind data of a particular area) instead of underlying physics because complex factors available in the data are directly incorporated into the models.

WTPC models have been classified into two groups in the literature: parametric and non-parametric methods [15]. The parametric methods use mathematical expressions to represent the relationship between wind speed and wind power, which can be characterized by a sigmoidal (s-shaped) curve [11, 16]. The commonly used parametric techniques are: modified hyperbolic tangent (MHTan) models [17, 18], polynomial models [19], ideal power curves [20], dynamic power curves [21], probabilistic models [22], parameter logistic functions [23], exponential models [16], etc. However, the performance of parametric methods is limited as they cannot describe the power curves very well because these methods are highly constrained to the specified functional form [18].

Non-parametric methods have an advantage over parametric methods because they are not based on a mathematical model and do not require previous knowledge about the shape of the power curve. Instead, they use real wind turbine data to model the power curves. For instance, Pelletier et al. developed an artificial neural network (ANN) using a multi-stage modeling technique which he used to model a power curve having six inputs including wind speed [24]. Ciulla et al. also developed a model based on multi-layered artificial neural networks (ANN) that predicted the generated power [25]. The actual power curve was then compared with the theoretical power curve and their variations were studied. In [26], the modeling performance of several techniques, including *k*-nearest neighbor (*k*-NN), adaptive neuro-fuzzy interference system (ANFIS), cluster center fuzzy logic (CCFL), and neural network, was compared. Tinghui et al. used the support vector machines (SVM) technique to build a WTPC model based on data mining and data partitions [27]. Lydia et al. also presents a comparative literature study on parametric and non-parametric methods [16]. Although non-parametric models are more flexible due to their data-driven nature, their parameters lack interpretability because of their black-box-like structure. Also, they are more computationally complex [18].

The data quality must be good to obtain accurate power curves [28]. Hence, during the pre-processing stage, we must detect and remove the obvious outliers from our data which are present there for many reasons (as discussed in detail in Section “Data sets and experimental design”). The main characteristic of these outliers is that their recorded power is far away from the theoretical power curve for a given wind speed. To remove them from training data, many researchers have used different filtration methods [5, 17, 19, 27]. A man-made approach was used in [18]. Kusiak et al. constructed a power curve using the K-nearest neighbor (KNN) model and then they used the residual method and control charts to detect the outliers in the data [29]. Zhao et al. [5] introduced a data-driven approach that combines quartile and density-based clustering methods to remove inconsistent samples. It should be emphasized that there is no such filtering method that can eliminate all the outliers from our data, and there remain some hidden outliers that affect the accuracy of the WTPCs. Mehrjoo et al. [30] addressed the inconsistency of the data and proposed a balanced loss function to mitigate its impact. Wang et al. utilized asymmetric error characteristics in developing two new spline regression models, where the error terms were assumed to follow asymmetric distributions [28]. However, most WTPC models in the literature are trained without considering the adverse impact of these hidden outliers.

From the above discussion, we can conclude that there are four main challenges in obtaining accurate and reliable power curve models. The first challenge is to develop a pre-processing technique to effectively remove the obvious and visible outliers from the datasets before modeling the power curve. To improve the modeling accuracy, the second challenge is to mitigate the adverse effect of the hidden outliers on the WTPC modeling accuracy. In addition to mitigating the effect of hidden outliers, the third challenge is to find an effective modeling technique that can accurately model the wind turbine power curve. After developing accurate power curves, the last challenge is developing a method to detect and remove hidden outliers (using the modeled power curve).

Multivariate models are developed by including other atmospheric and turbine-related variables such as air density, wind direction, yaw angle, rotor speed, Blade pitch angle, etc. [31, 32]. Such as Olivier et al. used regression trees such as boosted trees, extremely randomized forests, and random forests for multivariate power curve modeling [33]. It has been observed that when augmenting the wind-speed based models by using additional inputs, the variance in the generated power is better accounted for and accuracy also improves. Hence, the main aim of this paper is to propose a novel strategy for WTPC modeling that can alleviate the adverse effect of the hidden outliers on WTPC modeling by developing multivariate models and using this strategy to select the most optimum WTPC modeling technique.

This paper’s contributions are summarized as follows:

- A pre-processing technique is designed to remove the most obvious and visible outliers in the data. And the presence of hidden outliers is proved by the asymmetry of the error distribution, which remains after pre-processing.
- The error distributions of both univariate (with single input) and multivariate (with multiple input parameters) models are analyzed. It is proved that the adverse effect of the hidden outliers on the WTPC modeling accuracy is reduced by developing multivariate models because their error distribution has higher peaks and lesser standard deviation, which shows that the errors are more converged to zero and there is less number of extreme outliers compared to the univariate case. Due to this reason, multivariate models achieve better results in terms of accuracy.
- MARS is proposed for WTPC modeling and is proven to be the best WTPC modeling technique because it achieves the best overall average rank (based on accuracy) among all other techniques and gives good results in both univariate and multivariate cases.
- It is proved that compared to the Gaussian distribution, the Laplace distribution better fits the error distributions. Hence, an outlier detection method is developed to detect and remove the hidden outliers by generating a 97% confidence interval around the modeled curve using the Laplace distribution.

## Asymmetric error distribution in WTPC modeling

**Fig 1:**
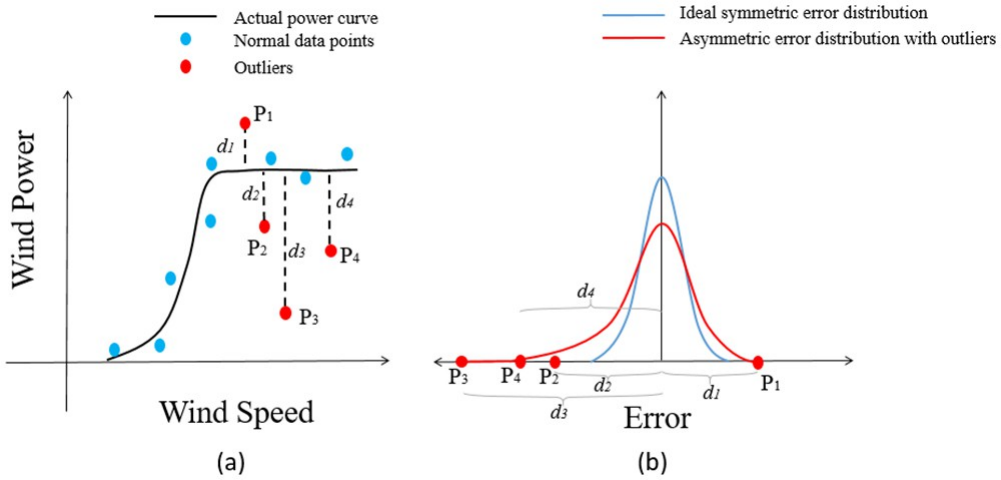
The effect of different outliers on the error distribution of the power curve.

Under normal conditions the measured wind power data is centered around a hidden power curve (actual power curve) that is the natural fit to the data, as shown in Fig 1(a). However, due to complex environmental conditions, climate changes and physical constraints of the wind turbine, numerous outliers are present in the measured power that are far from their corresponding ideal power on the actual WTPC [11]. The difference in measured and predicted wind power is the error in WTPC modeling. Fig 1 shows the effect of two types of common outliers on WTPC modeling [28, 34]. For a given wind speed, the measured power of outliers of the first type, such as P2, P3, and P4, is very small as compared to their corresponding (ideal) power on the actual power curve. As for the second type of outlier, denoted by P1, its measured power, is very high compared to its ideal value on the actual power curve, for a given wind speed. Hence, their distances (d1, d2, d3, and d4) are greater compared to the distances of normal data points from the power curve. In most cases, distances of the first type of outliers (d2, d3, and d4) from the actual power curve are greater than the distance of the second type of outliers (d1) due to the physical limitations of the wind turbine [28, 34]. Hence, the left tail of the error distribution curve of all the errors becomes longer than its right tail (left-skewed), which results in the error distribution being asymmetric, as shown in Fig 1(b). This phenomenon is also indicated by the negative value of skewness. As a result, the asymmetric error distribution of a power curve is an indication that there are outliers present in the training dataset. It is necessary to remove these outliers, otherwise they have a bad effect on the modeling accuracy [11]. To remove these outliers, many pre-processing techniques have been proposed [11]. However, these techniques can only remove the most visible and obvious outliers, but there remain some hidden outliers that cannot be removed through any pre-processing technique. This fact is also indicated later in Fig 10, where the error distribution of the power curve which is modeled using filtered data, is still asymmetric, indicating the presence of hidden outliers. Hence, a new modeling strategy is developed in this paper that can mitigate the effect of these hidden outliers on WTPC modeling.

## Wind turbine power curve models

There are many methods by which WTPC modeling is performed. In this section, a few popular parametric and non-parametric methods used in this paper, are introduced:

### Parametric models

#### Four Parameter Logistic Model (4-PLM)

The 4-PLM is widely used in wind power curve modeling because they follow a sigmoidal curve which is very similar to wind power curves. 4-PLMs are represented by (1):

$$y=\frac{a\left(1+be^{\frac{x}{d}}\right)}{1+ce^{\frac{x}{d}}}$$ (1)

$$\underset{a,b,c,d}{\text{min}}\overset{I}{\underset{i=1}{\sum }}{\left({\hat{\text{y}}}_{i}-y_{i}\right)}^{2}$$ (2)

Where x is wind speed and y is wind power (1). The shape of the wind power curve is determined by the four parameters *a*,*b*,*c*,*d*. These parameters are optimized using an intelligent optimization technique called least square error which assumes that minimizing the sum of the squared residuals will yield the best-fitting curve [17], as shown in expression (2), where ${\hat{\text{y}}}_{i}$ is predicted wind power and *y*_*i*_ is the real wind power and *I* is the total number of samples.

#### Five Parameter Logistic Model (5-PLM)

To approximate the shape of power curves, 5-PLM is used in [35]. It is represented by the expression (3):

$$y=a+\frac{b-a}{{\left(1+{\left(\frac{x}{c}\right)}^{d}\right)}^{e}}$$ (3)

Where *a*,*b*,*c*,*d*,*e* are the corresponding five parameters, where the range of *c*,*e* is: *c*,*e* ≥ 0. The same optimization technique that was used to tune the parameters of 4-PLM is used to optimize the parameters of 5-PLM as expressed in (2).

#### Polynomial regression

The wind turbine power curves have been modeled by polynomial regression in literature [2]. A *n*th degree polynomial is generally expressed as:

$$y=a_{0}+a_{1}x+a_{2}x^{2}+\ldots +a_{\text{n}}x^{n}$$ (4)

Where 0,…, *a*_n_, are the corresponding parameters that are also determined using the least-squares method. In practice, quadratic polynomials produce poor results, hence higher degrees of polynomials are used, with popular choices of 6-th and 9-th degree polynomial regression models (6PRM, 9PRM) for power curve modeling.

### Non-parametric models

#### Artificial Neural Network (ANN)

An Artificial Neural Network is a regression technique whose structure and working is similar to the structure of the neurons in our brain so that it can model the non-linear characteristics in the data very effectively. This is why ANN is widely used as a non-parametric modeling technique for WTPC modeling [36]. Backpropagation neural network (BPNN) is one of the most commonly used ANN technique, which can model any non-linear continuous function [37]. A BPNN, like every neural network, also has 3 layers: input, hidden, and output layers. BPNN uses the Gradient Descent Algorithm where backward propagation is used to adjust the values of weight and threshold of the network to achieve minimum sum-of-square error. The output of the network can be expressed as:

$$y^{o}=f\left(\sum_{n}^{N}w_{n}^{o}\;y_{n}^{h}+a^{o}\right)$$ (5)

Where *y*^*o*^ is the output of the network, which is obtained from an activation function (e.g. a linear function) *f*(.). Here, N represents the total number of hidden neurons, $w_{n}^{o}$ is the connection weight between nth hidden node (in the hidden layer) and the single output node [18], $y_{n}^{h}$ is the output of an nth neuron in the hidden layer, and *a*^*o*^ is the bias.

#### K-Nearest Neighbor (KNN)

The k-nearest neighbors (KNN) is a non-parametric technique (since it doesn’t make any assumptions on the data being studied) that can be used for regression as well as classifications. The K closest training samples in the dataset are the inputs of the KNN algorithm. The output of KNN in the case of regression depends on the values of K nearest neighbors. Using the known data parameters of instance x (wind speed), the unknown value of instance y (wind power) is predicted. The KNN does this by mapping the observations into multidimensional space and then classifying the observations in the clusters within a Euclidean distance k which is kept minimum [38]. For a training dataset of *N* instances of y and x, the distance between y and x is given by:

$$d\left(y,x\right)={\left({\left|\sum_{n=1}^{N}y_{n}-x_{n}\right|}^{a}\right)}^{\frac{1}{a}}$$ (6)

Where the number of instances of x and y is denoted by *N* and *a* is the order between them. The algorithm assigns weights to individual instances at this distance using the following expression (7):

$$w_{n}={\left(\frac{2\left(d+4\right)}{d+2}\right)}^{\frac{d}{d+4}}k$$ (7)

Here k represents the kernel function. The value for the unknown instance y is calculated by averaging the closest k neighbors from the training data.

#### Support Vector Machine (SVM).

The SVM is a non-parametric, supervised learning technique that analyzes data for both regression and classification analysis [27, 39]. In this model, a non-linear function is used to map input space into a high-dimensional feature space, by which the problem can be processed in linear form. SVM having ε-nonsensitive loss function (ε−SVM) is one of the most widely used SVM models for regression.

## Techniques introduced for WTPC modeling

In this section, MARS is proposed for WTPC modeling along with regression trees. Although some regression trees have been used for WTPC modeling in the literature, for example in Reference [33]. The four mentioned below are the most widely used Regression tree techniques, which are employed together for the first time in this study.

### Regression trees

Tree-based algorithms are one of the most widely used non-parametric models because of their high accuracy, stability, and simplicity. Furthermore, they can adapt to any kind of problem and can model non-linear relationships very well [33]. Some of the most important tree-based regression models which are used in this study are:

#### Decision tree regression (DTR)

The decision tree is a type of supervised learning method that uses a tree-like model of decisions and their possible outcomes. They express conditional control statements in a tree-like manner. Decision trees basically divide the samples into two or more homogeneous sets of populations based on the most significant differentiator among the input features [40]. The decision trees identify the most significant differentiator variable and its value that can give the best set of homogenous subsets of the population. A new prediction based on the decision tree can be calculated by going down the tree and taking the average value of the remaining samples in the subset.

In the decision trees, the root node is at the top, which represents the entire samples/population, the decision node is a sub-node that gets split into further sub-nodes (meaning that a decision can take place at this node), and the leaf node is a terminal node which does not split further.

#### Random forest regression (RFR)

Random forest is an ensemble learning method, that builds many decision trees during the training time. Like decision trees, the trees are constructed in a decision-outcome manner, in which the data is optimally split into subsets based on a specific selected feature. However, the major disadvantage of decision trees is overfitting. That is, they tend to learn the data itself instead of learning the underlying patterns in the data. Random forest is the solution to this problem because they use an ensemble method that creates regression trees using the bootstrap aggregation method (bagging) [41].

Bagging is a method of building multiple models using the same algorithm by repeatedly selecting different subsets of the dataset called bootstrap sampling. Also, in addition to the subsets of data, it also ensures that different features are used while training different trees. Hence, results are extracted in random trees by averaging the predicted values of each tree. As each tree is constructed using different portions of data and features, they are unlikely to over-fit.

#### Extra tree regression (ETR)

Similar to random forests, extra trees or extremely randomized trees add the aspect of randomization while building the trees [42]. The main difference is that the trees are trained using the whole learning dataset instead of the dataset’s subsets, and the splitting of the tree is randomized. At each step, rather than finding the best feature and its value for splitting the data, a random value is chosen for each feature. Extra tree regression has a low computational cost compared to random forests because the best possible split is found considering every feature and its corresponding values in RFR, however, in ETR, for every available feature, a random value is chosen.

#### Gradient boosting regression (GBR)

In RFR and ETR, each individual tree is independent of one another; however, they are built at the same time separately. Unlike these methods, gradient boost creates trees one by one in a forward iterative manner. Initially, the first tree is built on the learning dataset. The second model learns from the same dataset and the previous model’s errors. The third model also learns from the same dataset and errors of the second model. In this way, a number of models are built to gradually decrease the residual errors. Since each model learns from the errors of the previous models, they tend to have higher accuracy as compared to random forests. Overfitting in gradient boost is often tackled by using a regularization term called “learning rate”, which controls the contribution of the additional trees [43].

### Multivariate adaptive regression splines (MARS)

MARS is a regression-based non-parametric method that automatically models the complex nonlinearities in the data using the spline function. MARS works by dividing the entire data region into subdomains, each of which is fitted with a linear regression line. In that region, the slope of the linear regression line remains constant, while it varies in other regions. Hence, MARS uses piecewise linear splines for local fitting and then selects the final model by applying an adaptive technique. For one-dimensional cases, the general form of MARS is formulated as:

$$f\left(x\right)=\;\alpha_{\text{0}}+\;\overset{M}{\underset{n=1}{\sum }}\alpha_{\text{n}}\;BSF_{\text{n}}\left(x\right)$$ (8)

where *f*(*x*) denotes the response variable’s predicted value, *α*_0_ shows the value of intercept, which is the mean of response values, *BSF*_n_(*x*) denotes the nth basis function, and *α*_n_ is its corresponding coefficient. A MARS model’s basic component is the hinge function. A basic hinge function is of the form max{0, *x* − *a*} or max{0, *a* − *x*} with a knot at *a*. The following expression shows the hinge function *max*{0, *x* − *a*}:

$$Hn\left(x\right)=\;\text{max}\;\left\{0,\;x-a\right\}=\left\{\begin{array}{c} x-a\;,\;\;\;\;\;\;\;\;x>a,\\ 0,\;\;\;\;\;\;\;\;\;\;otherwise \end{array}\right.$$ (9)

The constant *a* is called a knot. The hinge function contains zero value for its range where *x* < *a*, so it is used to divide data into disjointed regions. As a result, each one of these regions can be treated separately. The basis function, which consists of hinge functions can be of three types: the first type is a constant 1, which is the intercept; the second one is a hinge function, and the third type is a product of two or more such hinge functions. The MARS algorithm is divided into two parts:

#### The forward pass

In the forward pass, the algorithm initially starts with the constant intercept term, which is the mean of response values. Then the algorithm repeatedly adds pair of the basis functions to the model. But among many possible basis functions, at each iteration, it only adds those pairs, which reduces the overall residual sum of squared error the most. The newly added basis function contains a constant term that is present in the model and it is multiplied by a new hinge function. MARS selects the new hinge function by searching all the possible values of each variable for the knot of the candidate hinge function. Hence, it finds the best combination of variables and knots at each step in a brute-force manner that increases the model’s precision. The process of adding the terms continues until the change in the residual error is very low and insignificant or if the model reaches its maximum number of terms limit that the user initially sets before the training starts.

#### The backward pass

In most cases, the forward pass constructs an over-fit model, which generalizes well to the data it is trained on but shows poor results for an unfamiliar dataset. Thus by pruning the model, the backward pass improves its generalization ability. It means those basis functions that have the least contribution to the model’s prediction ability are removed at each step until the algorithm finds the best sub-model. The backward pass compares the performance of each model subset using generalized cross-validation (GCV) in order to choose the best subset model. The model with the lowest value of GCV gets chosen as the optimal model. GCV is a kind of regularization that balances model complexity and goodness of fit. The value of generalized cross-validation (GCV) is calculated from the following formula (10):

$$GCV=\frac{1}{O}\frac{RSST}{{\left(1-\frac{CC\left(B\right)}{O}\right)}^{2}}$$ (10)

$$CC\left(B\right)=B+1+pB/2$$ (11)

where *O* is the total number of observations, *RSST* is the residual sum of squares that is measured on the training dataset. The term *CC*(*B*) is the complexity cost function, given by Eq (11). *B* is the number of variable basis functions, and *p* is the coefficient of penalty, of which value is mostly used between two and five.

## Data sets and experimental design

### Data description

Two real-world datasets from different wind turbines were used in this study with 10-minute interval data each. Both datasets were recorded using the SCADA system. Dataset one (DS1) contains 50530 values that were recorded from a wind turbine in turkey and contains data from January to December 2018 (one year). Dataset two (DS2) has 118224 values from January 2018 till March 2020. Both datasets contain values of wind speed, generated wind power, and wind direction. However, DS2 contains values of a few other parameters as well. For DS1, 32120 values are used for training, and 15820 are used for testing, while for DS2, 38978 are used for training, and 19198 are used for testing purposes as shown in Table 1. The software platform is Python 3.6.9. The hardware system for the experiments has 12 GB RAM single-core hyper threaded Xeon Processors (2.3Ghz).

**Table 1:** The number of samples in training and testing set after pre-processing.

Dataset	Samples	Missing data points	Outliers	Training Set	Testing Set
DS1	50530	0	2590	32120	15820
DS2	118224	46062	13986	38978	19198

### Data-preprocessing

**Fig 2:**
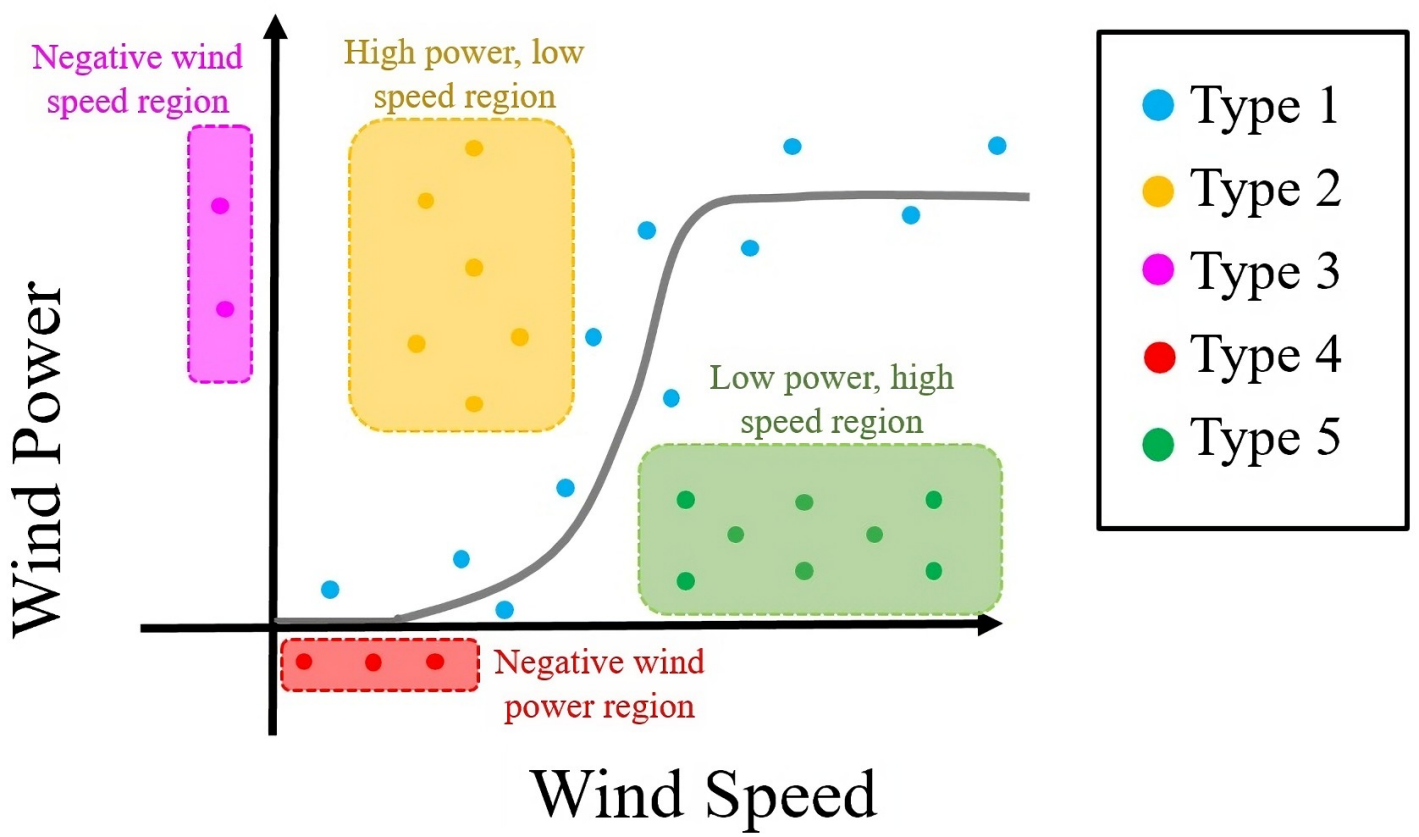
Classification scheme of different outliers in wind power data.

There are many reasons for the presence of outliers in any unfiltered wind dataset. It is necessary to find and remove these outliers using any data pre-processing technique; otherwise, the outliers’ presence could adversely affect WTPC modeling accuracy. Liu et al. have classified wind power data into five types, as shown in Fig 2 [44]. According to this approach, the normal data which is obtained when the turbine works in normal conditions are represented by the first type, and the rest of the types are different outliers. Among these five types, the second type of data points has high wind power for low wind speeds, which can be caused by mechanical problems in the wind turbine, faults in the communication channels, or disability of the sensors. The third type is the data samples, where wind speed has negative values. The outliers of this type is likely caused by a calibration error in an anemometer. The fourth data type has negative wind power values because, in this case, the power generated by wind turbines is insufficient to meet the turbine’s own electrical needs. Low wind power at high wind speeds characterizes the fifth type of data sample, which can be caused by strong winds from the wrong direction or if the turbine is not online at such high speeds. According to this classification scheme, all data types are present in both datasets used in this study (DS1, DS2), except the third type. Taking this consideration into account a new pre-processing approach is designed to remove the outliers.

**Fig 3:**
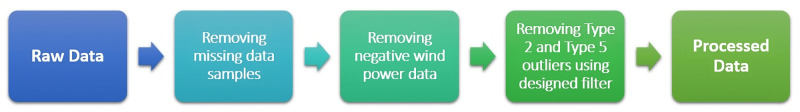
Main steps of pre-processing.

**Fig 4:**
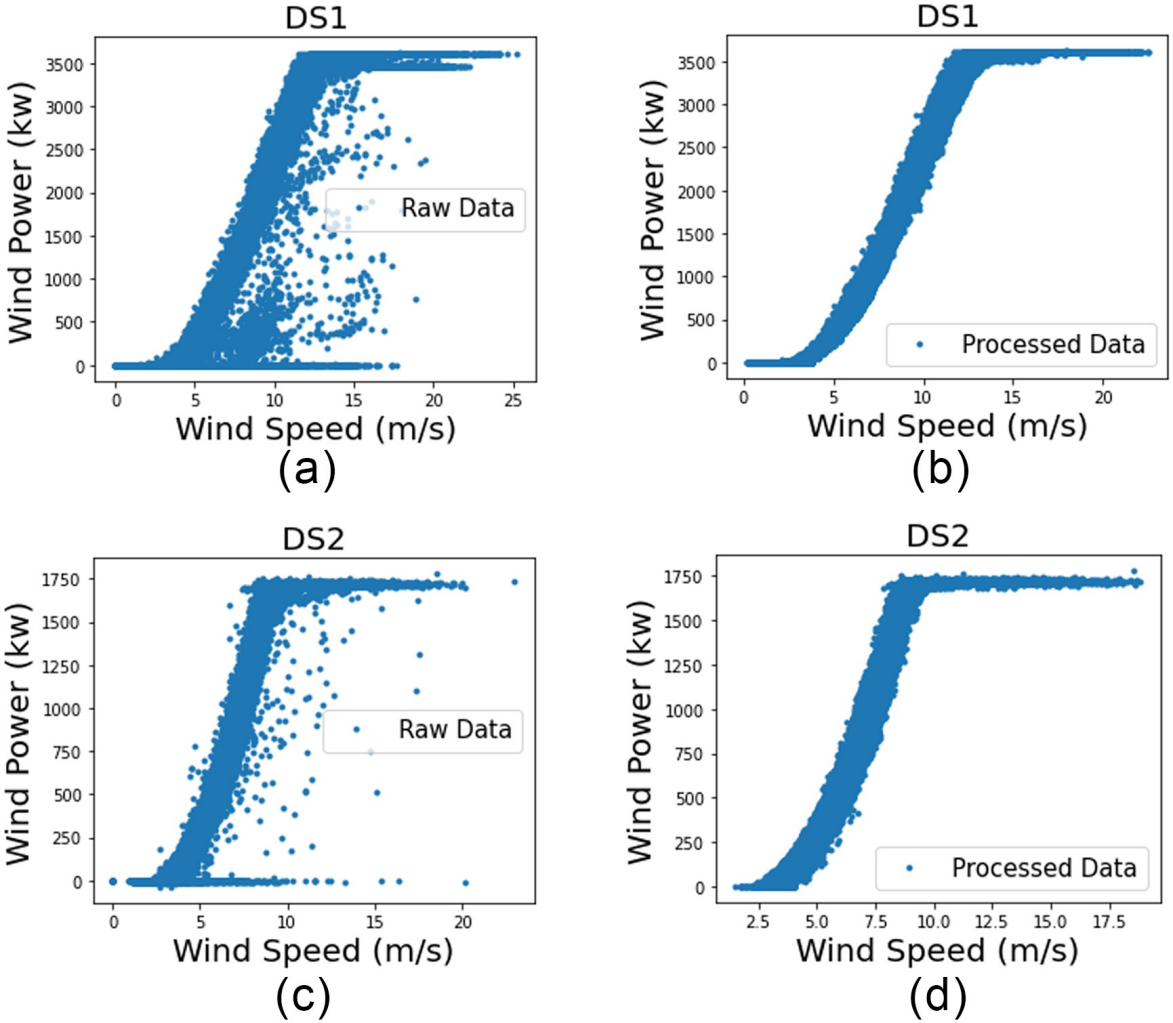
(a) and (c) show raw data for DS1 and DS2, respectively. (b) and (d) show pre-processing results for DS1 and DS2, respectively.

In this paper, pre-processing is done in three steps, as shown in Fig 3. In the first step, missing data points are removed from the raw dataset. DS2 has 46062 missing data values, while DS1 has none. The second step involves removing type four outliers, where negative wind power values are removed from the dataset. In the third step, type two and type five outliers must be removed. In this paper, a new method is used to remove these types of outliers, which can be clearly seen on the scatter plot of wind speed and its corresponding wind power in Fig 4(a) and 4(c). In this method, a filter is designed that works in two steps. In the first step, the wind power values are split into many small intervals called data frames. The number of these data frames is adjustable depending on the scatter plot of the dataset. To get fine results, the number of data frames is increased. In this paper, DS1 and DS2 are divided into 74 and 58 data frames, respectively. In the second step, for each data frame, a range of data points is selected as normal data points based on their wind speed values, and visible outliers with extremely low and extremely high wind speed values are removed. This is done by setting a lower and higher limit of the threshold for wind speed values in each data frame. The values of these threshold limits are controlled through the quantiles of the data present in the data frame, which are adjusted through trial and error so as to remove all the obvious outliers. Hence, the upper and lower limit of the threshold is set by us. However, we have kept these limits considerably flexible so that only visible outliers can be removed. Hence, there remains a possibility that some hidden outliers might still remain in the pre-processed training dataset. This filtering method is better than the other rigid interval-based methods discussed in [11, 28, 45] because of its flexibility in adjusting the number of data frames and values of the low and high threshold for each data frame, which gives better control to remove the outliers. The result of this pre-processing technique on the raw data of both datasets is shown in Fig 4.

### Evaluation indices

In this paper, the error indices which are used to evaluate the performance of all the models include, Mean Absolute Error (MAE) [18], Root Mean Square Error (RMSE) [2], and R-squared Error (R^2^) or the coefficient of determination [28], which are computed as:

$$MAE=\;\frac{1}{N}\sum_{n=1}^{N}\left|y_{n}-\;{\hat{\text{y}}}_{n}\right|$$ (12)

$$RMSE=\;\sqrt{\frac{1}{N}\sum_{n=1}^{N}{\left(y_{n}-\;{\hat{\text{y}}}_{n}\right)}^{2}}$$ (13)

$$R^{2}=1-\frac{\sum_{n=1}^{N}{\left(y_{n}-\;{\hat{\text{y}}}_{n}\right)}^{2}}{\sum_{n=1}^{N}{\left(y_{n}-\;\bar{\text{y}}\right)}^{2}}$$ (14)

The N represents the number of test samples. *y*_*n*_, ${\hat{\text{y}}}_{n}$ represent the actual (measured) and predicted wind power, respectively and $\bar{\text{y}}$ is the average of the actual wind power. These evaluation indices are chosen based on their demonstrated effectiveness in regression techniques, particularly in WTPC modeling literature [33, 35, 46].

For each model, lower values of the MAE and RMSE indicate higher accuracy, and R^2^ values closer to 1, show the increase in the goodness of fit of the power curve.

## Results and discussion

### Hyper-parameter tuning

In this paper, four parametric (4-PLM, 5-PLM, 6-PRM, and 9-PRM) and three learning-based non-parametric methods (BPNN, KNN, and SVM) are used as the benchmark against four tree-based models (DTR, RFR, ETR and GBR) and MARS. The optimal hyper-parameters are needed to achieve maximum performance on training these methods on the training data in a reasonable amount of time. For parametric methods, their optimal parameters are determined using the least square optimization method [2]. In order to obtain the best parameter estimates, the least squares method minimizes the sum of the square of residuals. The structure of the BPNN drastically affects the modeling results. The Hecht-Nielsen method [47] that determines the number of layers and neurons, used in [18], does not produce the best results. Hence BPNN with 2 hidden layers is chosen in this paper, which is also used in [24]. As BPNN gives different results for the same parameters each time it is trained because of the random values of gains and weights in its structure, an approach based on trial and error is used in finding the number of layers (for which two are selected) and the number of neurons per layer. By experiments, trends in the results are determined and parameters, where prediction error converges, are explored further. For SVM, RBF kernel is the best option for kernel. The best value for regularization parameter C and kernel coefficient gamma is found through an exhaustive grid search method. For KNN and all regression tree methods (DTR, RFR, ETR and, GBR), the Bayesian Optimization (BO) technique with 5-fold cross-validation (using “BayesSearchCV” package of the scikit-optimize library of python) is used to find the best combination of the hyper-parameters on the training set [48]. The BO algorithm is a powerful global optimization technique based on Gaussian processes and Bayesian inference. 5-fold cross-validation is chosen through experimentation, keeping in view the trade-off between finding lesser prediction error (higher accuracy) and greater convergence speed. 5-fold cross-validation means that the training dataset is split into 5 pieces, where one piece is used for testing and 4 remaining pieces are joined together and used to train the model for a specific combination of the hyper-parameters. This process is repeated until all 5 pieces are used for testing. To ensure generalization the original testing dataset is not used in hyper-parameter optimization [49]. The most optimal hyper-parameters are then used to train the models using all the training data set for wind power curve modeling.

The training time MARS takes depends on the threshold value given to one of its parameters, which governs the stopping conditions for the forward pass. The lesser its value, the greater forward pass will be. In this paper, the value of the threshold is set very low (between 3E-7 to 3E-6), especially in multivariate cases, to increase the forward pass as much as possible for the MARS model to have many basis functions to choose from in its pruning pass, because it results in lesser prediction error, which increases the time it takes to train the model drastically. Hence the same experimental approach is used here which was used for BPNN. Table 2 presents optimal hyper-parameters of all methods for DS1 when only one input is given (univariate case). For ease of presentation the default parameters of each method is not shown. The parametric methods are implemented by us, however, non-parametric methods are implemented using their available python packages.

**Table 2:** The estimated parameters of power curve models modeled using DS1 for univariate case.

Model	Optimal Hyper-parameters
4PLM	a = 3701.61, b = -3.95, c = 1.64, d = 199.76
6PRM	a = -311.30, b = 508.99, c = -265.49, d = 57.21, e = -4.84, f = 0.17, g = -0.0024
5PLM	a = 3640.56, b = -14.67, c = 86.25, d = 3.80, e = 4181.75
9PRM	a = -107.59, b = 307.26, c = -284.68, d = 118.30, e = -26.34, f = 3.66, g = -0.31, h = 0.015, i = -0.00039, j = 4.28e-06
ETR	n_estimators = 2800, max_depth = 500, min_samples_split = 2, min_samples_leaf = 1, max_leaf_nodes = 95
DTR	max_depth = 397, min_samples_split = 7, max_leaf_nodes = 97, min_samples_leaf =
GBR	learning_rate = 0.099, n_estimators = 100, max_depth = 10, min_samples_split = 20, min_samples_leaf = 451, max_leaf_nodes = 77
BPNN	hidden_layer_sizes = (310,310), max_iter = 1000
KNN	n_neighbors = 900, leaf_size = 38, p = 5, weights = ‘uniform’
RFR	n_estimators = 51, min_samples_split = 47, min_samples_leaf = 92, max_depth = 52, max_leaf_nodes = 800
MARS	max_degree = 1000, max_terms = 1200, thresh = 0.000001, minspan = 1, penalty = 1,
SVM	kernel = ’rbf’, C = 300, gamma = 0.5

### Univariate power curve modeling

**Fig 5:**
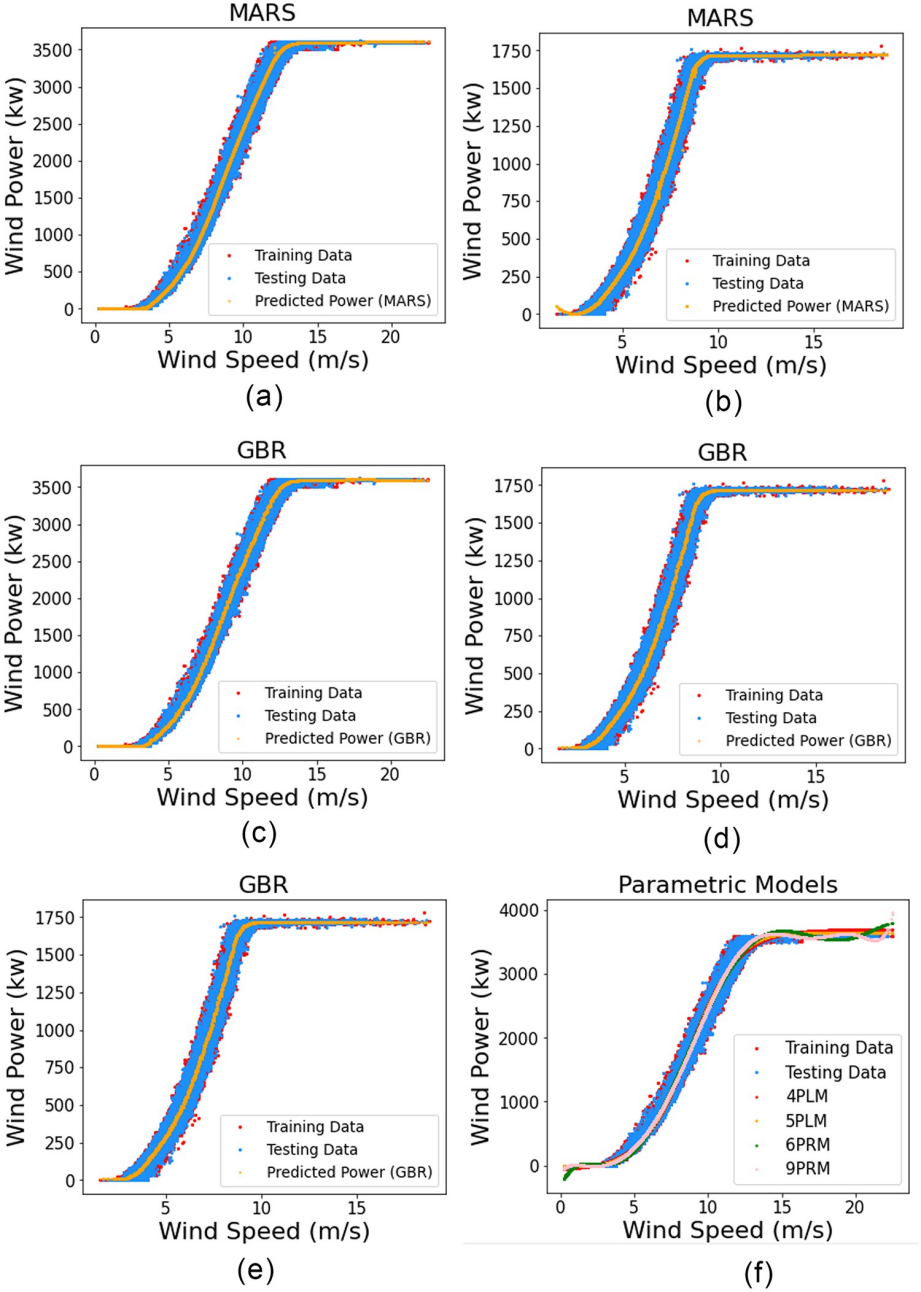
(a) (c) and (e) show Univariate Wind Power Curves of MARS, GBR and Parametric models for DS1, respectively. (b) (d) and (f) show Univariate Wind Power Curves of MARS, GBR and Parametric models for DS2, respectively.

In this section, WTPC modeling is done using both parametric and non-parametric models with only one input (wind speed) and their performance is compared. The results of univariate power curve modeling of all algorithms are presented in Table 3 and the rank of their performance is given in Table 4. The estimated univariate power curves of GBR, MARS and parametric models are shown in Fig 5.

**Table 3:** The result of univariate power curve modeling of different models.

Model	DS1			DS2			Ave. Time (s)
	MAE(kW)	RMSE(kW)	R^2^	MAE(kW)	RMSE(kW)	R^2^	
4PLM	73.9413	104.7571	0.994187	60.4838	77.9763	0.982430	**0.0031**
6PRM	71.8633	101.5121	0.994542	58.5772	77.0370	0.982851	0.0147
5PLM	64.8941	98.0978	0.994903	55.7715	73.2526	0.984494	0.0074
9PRM	60.8008	95.2226	0.995197	52.3003	70.4332	0.985665	0.0262
ETR	57.3729	94.5060	0.995241	46.4158	65.6766	0.987510	7.9747
DTR	57.3606	95.0819	0.995183	46.1418	65.7048	0.987500	0.0386
GBR	57.1166	94.6523	0.995226	46.0094	65.4857	0.987583	2.9581
BPNN	57.0100	95.0614	0.995185	45.9762	65.3516	0.987634	80.500
KNN	57.0465	94.5097	0.995241	45.9559	**65.2940**	**0.987655**	0.0115
RFR	57.0337	94.6337	0.995228	45.9402	65.4101	0.987611	1.7406
MARS	57.0745	**94.4897**	**0.995243**	45.8896	65.3404	0.987638	1258.1
SVM	**56.1177**	95.1692	0.995174	**45.6111**	65.5314	0.987565	111.24

**Table 4:** Ranking of performance of different models for univariate case.

Model	DS1			DS2		
	MAE(kW)	RMSE(kW)	R^2^	MAE(kW)	RMSE(kW)	R^2^
1	SVM	MARS	MARS	SVM	KNN	KNN
2	BPNN	ETR		MARS	MARS	MARS
3	RFR	KNN	KNN	RFR	BPNN	BPNN
4	KNN	RFR	RFR	KNN	RFR	RFR
5	MARS	GBR	GBR	BPNN	GBR	GBR
6	GBR	BPNN	9PRM	GBR	SVM	SVM
7	DTR	DTR	BPNN	DTR	ETR	ETR
8	ETR	SVM	DTR	ETR	DTR	DTR
9	9PRM	9PRM	SVM	9PRM	9PRM	9PRM
10	5PLM	5PLM	5PLM	5PLM	5PLM	5PLM
11	6PRM	6PRM	6PRM	6PRM	6PRM	6PRM
12	4PLM	4PLM	4PLM	4PLM	4PLM	4PLM

The results indicate that for both datasets, data driven non-parametric techniques perform much better than the parametric techniques. Among the parametric methods, 9PRM gives the best results, where in the case of DS1, according to R^2^, 9PRM takes the 6^th^ rank, however in all other cases parametric techniques shows bad results compared to non-parametric techniques.

**Fig 6:**
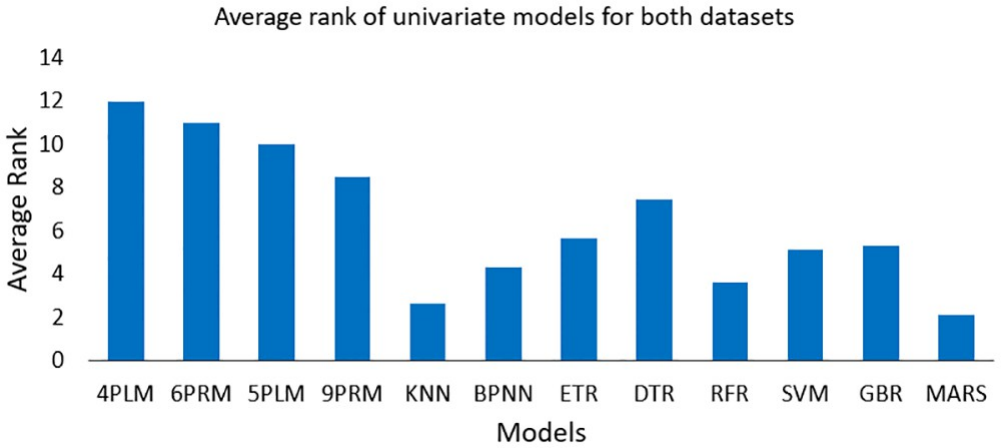
Average rank of all the models for univariate case, including rank of all error indices for both datasets.

For univariate power curve modeling, MARS gives the best result. In order to measure the overall performance of all univariate WTPC models, the average of their ranking (as shown in Table 4), for all three error indices including both datasets, are taken and compared. Fig 6 shows the average ranking of all models. According to Fig 6, MARS achieves the best average rank of 2.1, compared to all other models. For DS1, in case of MAE SVM gives the best result, for RMSE and R^2^ MARS provides the best results. For DS2, MAE indicates SVM as the best model, RMSE and R^2^ show that KNN gives the best results. Although SVM gets the best results according to MAE for both datasets but for other indices it gets ranked as low as 9^th^, hence on average its rank is 5.1 which is very low. KNN performs relatively better than SVM but not as good as MARS, with an average rank of 2.6. Among the regression trees only, Random Forest (RFR) gives good performance with an average rank of 3.6, compared to other tree-based methods. Overall, tree-based techniques do not perform very well in univariate cases.

Although results from univariate case give useful insights into the performance of different techniques but in this case information from only one input variable gets incorporated into the models. However, in the next section, one more input variable is used, which provides additional useful information for models, and then their performance is observed.

### Multivariate power curve modeling

In this section, in addition to wind speed, wind direction is also included as an additional input to all the models. Although we have many other input variables in DS2, only wind speed and direction are available in DS1. Hence the limitations of our datasets force us to use only two inputs for multivariate modeling in this paper.

**Fig 7:**
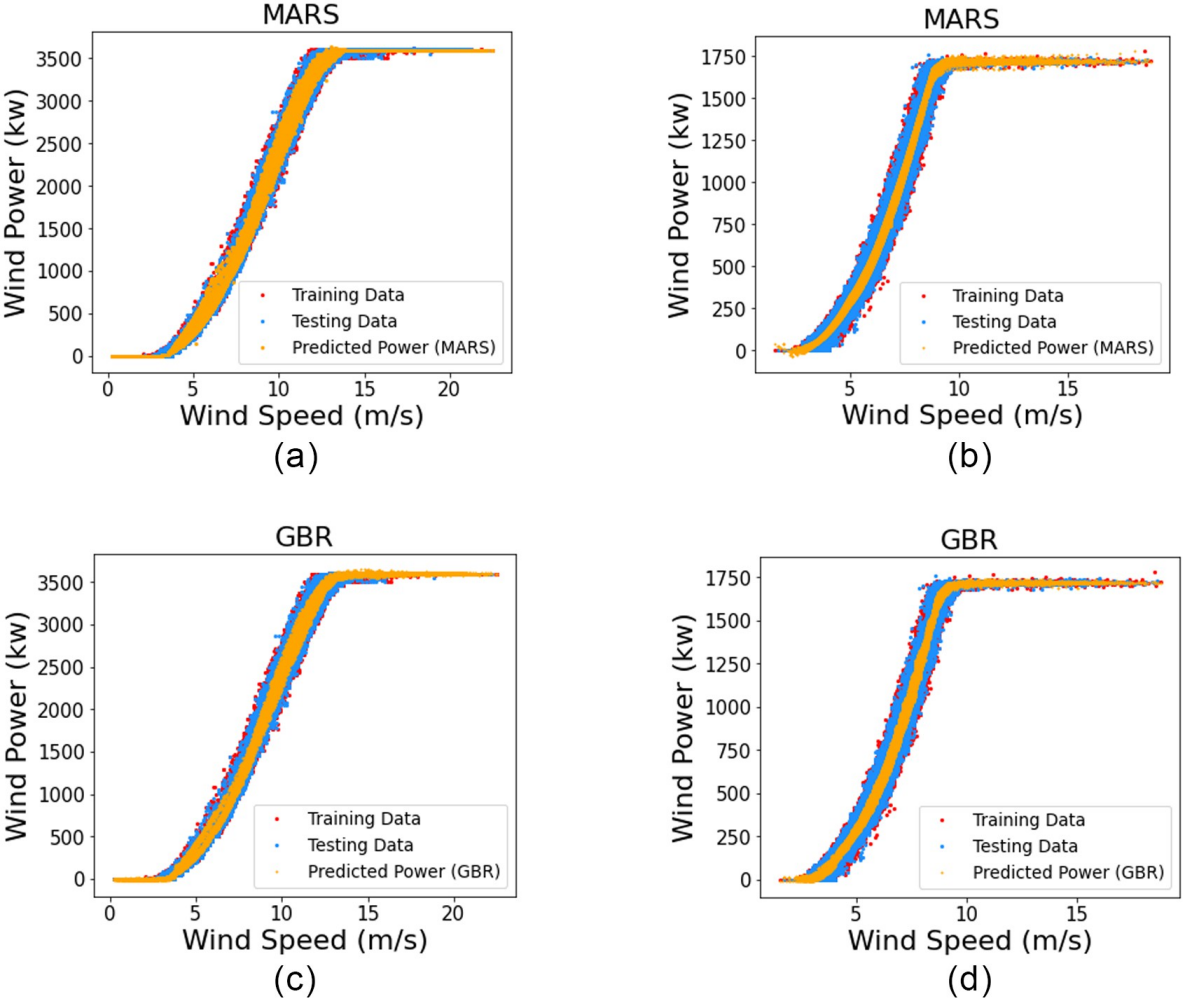
(a) and (c) show Multivariate Wind Power Curves of MARS and GBR for DS1, respectively. (b) and (d) show Multivariate Wind Power Curves of MARS and GBR for DS2, respectively.

**Fig 8:**
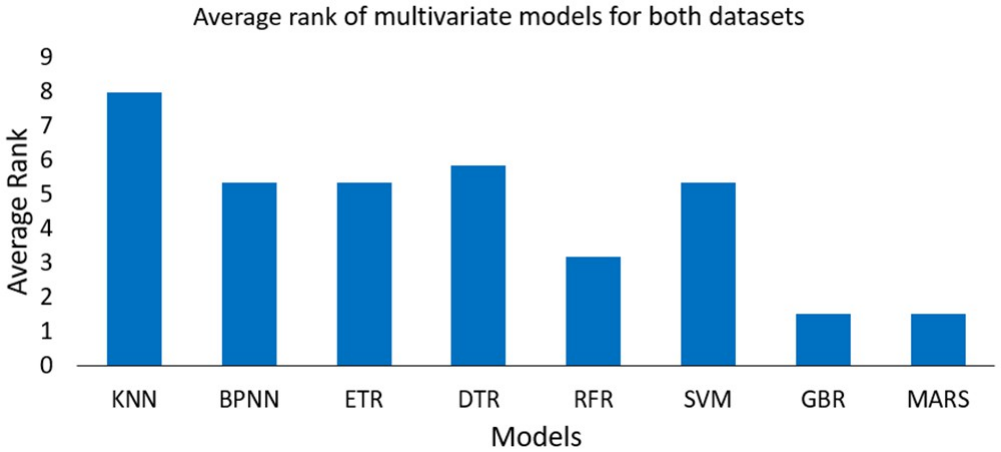
Average rank of all the models for multivariate case, including rank of all error indices for both datasets.

It is observed that MAE and RMSE decrease significantly compared to the univariate case, and overall results are improved. For instance, in case of MAE, MARS shows an improvement of 9.4% for DS1 and 4.94% for DS2. This is because, for multivariate case, the WTPC model takes into account information from two variables instead of one. Also, the variance of the predicted power improves and is better accounted for compared to the univariate case, as shown in Fig 7. It clearly indicates that the multivariate power curves give a better representation of our dataset, which results in better prediction results. As parametric techniques cannot be applied in cases with more than one input, hence they are excluded from the multivariate comparison of models. The results of non-parametric techniques for a multivariate case are shown in Table 5, and comparison of their respective rankings is shown in Table 6. For DS1, MARS gives the best results, and GBR takes the second rank in performance, according to all three error indices. In the case of DS2, GBR provides the best results, while MARS comes in second. Fig 8 shows the average ranks of each multivariate models, taking into consideration their rank for all error indices for both datasets, which are shown in Table 6. Although the ranking position of both GBR and MARS is different in both datasets, but their results are almost the same w.r.t. MAE and RMSE, hence they both achieve the same average rank of 1.5 for the multivariate case.

**Table 5:** The result of multivariate power curve modeling of different models.

Model	DS1			DS2			Ave. Time (s)
	MAE(kW)	RMSE(kW)	R^2^	MAE(kW)	RMSE(kW)	R^2^	
KNN	60.1723	102.7888	0.994370	48.2231	71.5224	0.985188	**0.0170**
BPNN	57.7711	89.7158	0.995711	45.2336	63.9791	0.988148	119.97
ETR	56.3110	92.2585	0.995465	44.6114	63.5622	0.988301	5.6954
DTR	54.5925	90.8177	0.995605	44.8478	64.2148	0.988060	0.0665
RFR	53.1039	88.5888	0.995818	44.2197	63.4234	0.988352	12.621
SVM	52.1352	90.1205	0.995672	44.7633	65.1275	0.987707	470.24
GBR	51.9717	85.7586	0.996081	**43.2638**	**62.3613**	**0.988739**	12.122
MARS	**51.3499**	**85.4395**	**0.996110**	43.6186	62.6752	0.988626	4962.5

**Table 6:** Ranking of performance of different models for multivariate case.

Model	DS1			DS2		
	MAE(kW)	RMSE(kW)	R^2^	MAE(kW)	RMSE(kW)	R^2^
1	MARS	MARS	MARS	GBR	GBR	GBR
2	GBR	GBR	GBR	MARS	MARS	MARS
3	SVM	RFR	RFR	RFR	RFR	RFR
4	RFR	BPNN	BPNN	ETR	ETR	ETR
5	DTR	SVM	SVM	SVM	BPNN	BPNN
6	ETR	DTR	DTR	DTR	DTR	DTR
7	BPNN	ETR	ETR	BPNN	SVM	SVM
8	KNN	KNN	KNN	KNN	KNN	KNN

It can be observed from the results in Tables 5 and 6, that the position of tree-based methods greatly improves compared to the univariate case, with GBR achieving the best average rank (same as MARS) and RFR giving the second-best average rank (with an average rank of 3.1), as shown in Fig 8. Compared to the univariate case the rank of SVM and BPNN considerably drops for multivariate case. The KNN model gives the worst performance among all the models for multivariate case. This is because the dimensionality of the input feature space increases for multivariate case. Hence, the number of K neighbors decreases to get better results which makes the KNN model more sensitive to the outliers.

**Fig 9:**
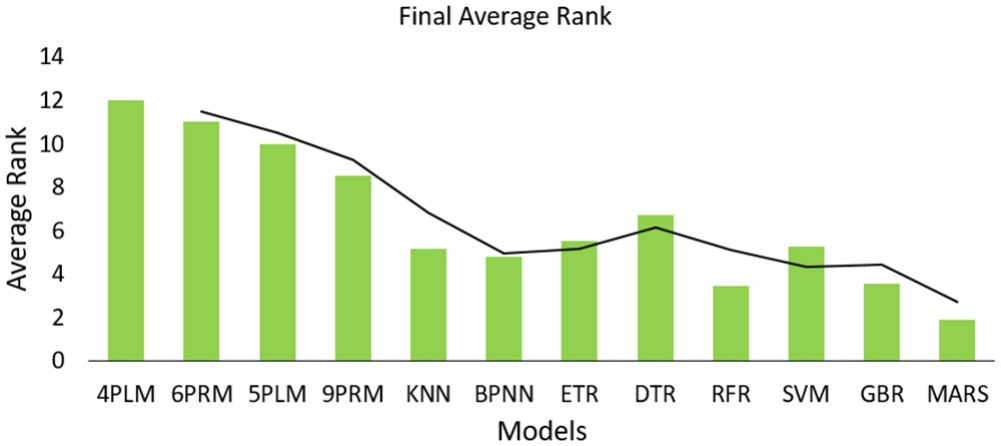
Overall average rank of all the models for both univariate and multivariate cases for both datasets.

The comparison of univariate and multivariate results indicates that among all models, MARS is the only technique that gives good results in both univariate and multivariate cases. Fig 9 shows the overall average of ranks for both univariate and multivariate cases for all the models. It can be proved through Fig 9 that MARS is the best technique for WTPC modeling among the other techniques used in this paper, as it achieves the best overall average rank, including all the cases of both univariate and multivariate results. Unlike MARS, some non-parametric methods give good results in a univariate case like SVM and KNN and some works well in a multivariate case like GBR and RFR but do not perform very well in both cases. In Fig 9, the final average rank of all models is presented, which shows that MARS achieves the best overall rank (final average rank of 1.8), RFR comes in second with a final average rank of 3.4, while GBR comes in third with a final average rank of 3.7.

Machine learning models can produce different outcomes for different error indices due to differences in sensitivity to different aspects of model performance, weighting of error indices, and statistical qualities of the data being evaluated. Due to this reason, many studies related to WTPC modeling use average ranking of their models for different error induces to evaluate their performance. Although the MARS model could not obtain the lowest error for all error indices, it did achieve the best average rank across multiple error indices. This demonstrates its competitive ability in capturing the complex relationships found in wind power data. Additionally, MARS models provide interpretability by describing the relationship between input variables and the target variable. This characteristic is extremely useful in WTPC modeling since it allows us to understand the underlying factors affecting power generation and obtain practical insights for operational optimization. At the same time, the training time for MARS model is higher than other models because it is given more iteration to be fully trained. The higher training time is justifiable because it is necessary to ensure that model captures the complex underlying patterns effectively and provides improved accuracy.

It can be observed that the WTPC in the univariate case is a simple line curve, as shown in Fig 5 where each value of wind speed has a single corresponding value of wind power. However, for the multivariate case, the power curve is like a plane instead of a simple line, as evident from Fig 7, where for each value of wind speed there is a range of wind power values. This is due to the fact that in the multivariate case, at the same value of wind speed, there are multiple different values of wind direction which results in multiple values of predicted wind power for a corresponding wind speed value. Hence, the variance is improved, and the modeled wind power curve is a better representation of our dataset.

Although deep learning models have been extensively used for predictive modeling tasks in the literature [50, 51], however they are less suitable for curve modeling (especially univariate WTPC modeling) because they require larger amounts of data and more computational power for training compared to traditional machine learning models. Hence, they have a potential for multivariate WTPC modeling, where many input features (which were unavailable in dataset 1 used in this study) are available in all the training datasets.

### Error distribution analysis of the trained models

**Fig 10:**
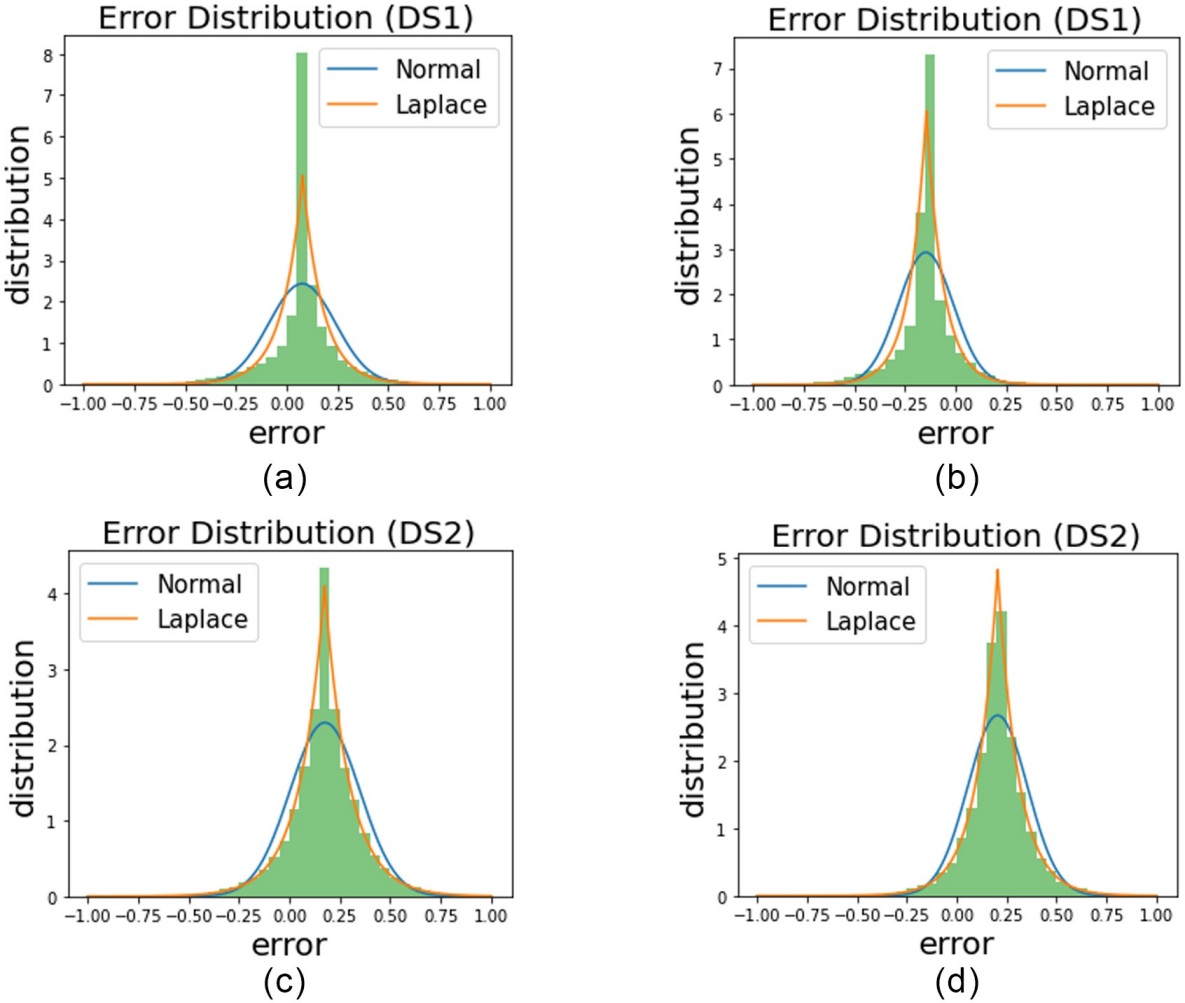
(a) and (b) show the Error Distribution of GBR for Univariate and Multivariate, respectively for DS1. (c) and (d) show the Error Distribution of GBR for Univariate and Multivariate, respectively for DS2.

During the pre-processing stage, the majority of the obvious outliers are removed from both datasets before modeling the power curve. However, some outliers cannot be detected by any filtering technique and these hidden outliers result in asymmetric error distribution of the estimated power curve as discussed in Section “Asymmetric error distribution in WTPC modeling”. The calculated error distribution (distribution of the error between actual and predicted wind power) of GBR for all four cases is presented in Fig 10, where the error distribution is represented through green histograms. We have used GBR because it shows considerable improvement from univariate to the multivariate case, hence it is easy to visualize the results. To analyze the characteristics of these distributions, some statistical measures are employed including skewness, kurtosis, and standard distribution.

Skewness is employed to measure the asymmetry of error distributions. For a Normal (or Gaussian) distribution, the value of skewness is 0, which means the distribution is symmetrical. In Table 7, values of skewness of the error distributions of MARS, GBR, KNN, and RFR are shown for all 4 cases. It can be seen for all cases in Table 7, that values of skewness are negative, suggesting that error distributions are asymmetrical and skewed left (having a longer left tail). This characteristic of the skewness verifies our conclusion in Section “Asymmetric error distribution in WTPC modeling”, as it proves the presence of hidden outliers.

**Table 7:** Skewness of error distributions of MARS, GBR, KNN, and RFR for both datasets and both cases.

Skewness	DS1		DS2	
	Univariate	Multivariate	Univariate	Multivariate
MARS	-0.4599	-0.3551	-0.1272	-0.1555
GBR	-0.4605	-0.3783	-0.1350	-0.1707
KNN	-0.4768	-1.2125	-0.1393	-0.8837
RFR	-0.4516	-0.4333	-0.1298	-0.1801

Kurtosis of a distribution is a measure of the peak of the distribution or, conversely how fat-tailed the distribution is. The kurtosis of a Gaussian distribution is 3. The values of kurtosis of the error distributions are presented in Table 8. It can be observed from Table 8 that the kurtosis values of all the multivariate models are greater than their corresponding univariate models. High kurtosis in distribution means that more of the variance is due to a lesser number of large deviations rather than the very frequent small deviations [52]. It means that an increase in kurtosis will lead to a lesser number of big outliers (which have a large deviation from the mean). The resulting shape of the distribution will have comparatively higher peaks and thinner but longer tails [52, 53]. An increase in the peaks suggests that the error points that were previously near the tails have moved toward the mean (which is zero in our case), and an increase in their frequency near the peak has increased the height of the peak.

**Table 8:** Kurtosis of error distributions of MARS, GBR, KNN, and RFR for both datasets and both cases.

Kurtosis	DS1		DS2	
	Univariate	Multivariate	Univariate	Multivariate
MARS	3.7843	4.4186	2.2756	2.8236
GBR	3.7919	4.2715	2.2758	2.9310
KNN	3.7686	9.1944	2.2545	4.8319
RFR	3.7783	4.0395	2.2664	2.6756

This means that the errors are more converged to zero and overall the number of outliers has been reduced. This effect is visible in Fig 10, where the error distributions of the multivariate models have comparatively higher peaks.

At the same time where kurtosis is increasing for the multivariate case, the standard deviation is decreasing as shown in Table 9. A decrease in the standard deviation of distribution means that there is a decrease in the number of extreme outliers [54], [55]. Hence, the side effect of the increase in the peaks, which is having longer tails (more extreme outliers), has been catered for. The combined effect of an increase in kurtosis and a decrease in the standard deviation of the multivariate distributions means that overall, the errors are more converged to zero and there are less number of extreme outliers compared to the univariate case, which results in lesser MAE and RMSE (as shown in Tables 3 and 5). This result proves that the adverse effect of the hidden outliers on the accuracy of WTPC modeling is reduced by developing multivariate models.

**Table 9:** Standard deviation of error distributions of MARS, GBR, KNN, and RFR for both datasets and both cases.

Standard Deviation	DS1		DS2	
	Univariate	Multivariate	Univariate	Multivariate
MARS	0.1627	0.1268	0.1710	0.1535
GBR	0.1641	0.1363	0.1738	0.1495
KNN	0.1632	0.1094	0.1708	0.1406
RFR	0.1634	0.1511	0.1725	0.1633

**Fig 11:**
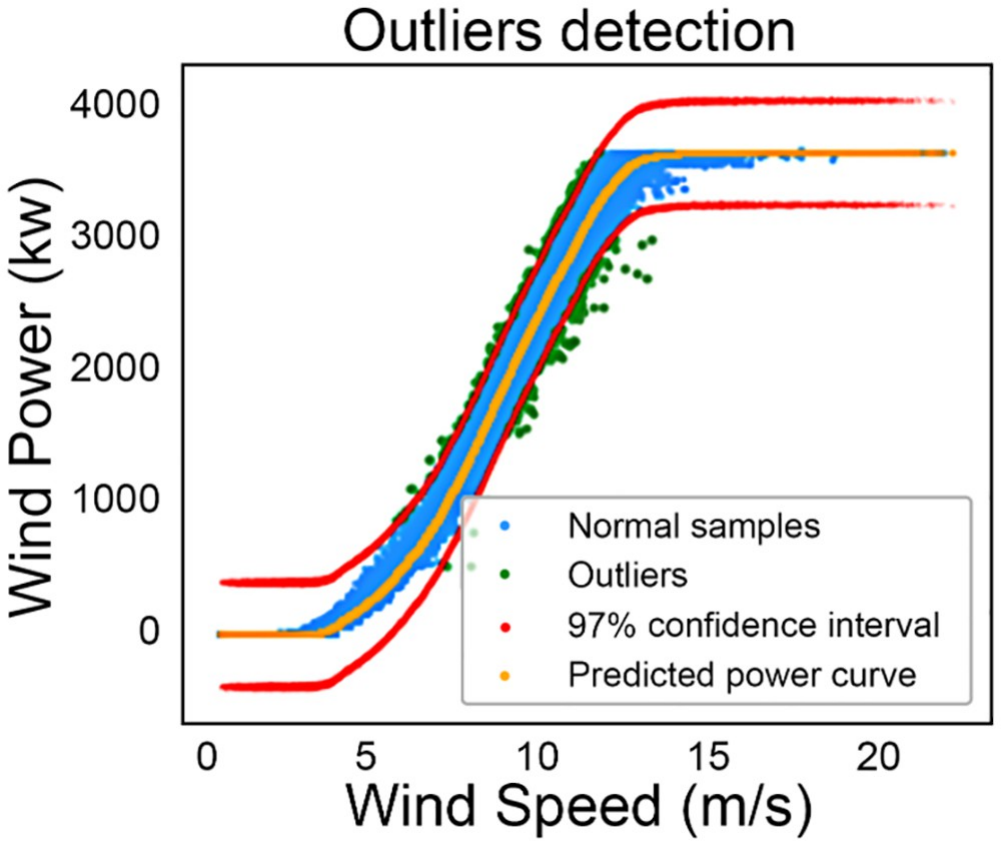
Outlier detection results on DS1 where confidence interval is built using error distribution of GBR.

It can be seen in Fig 10, that a normal distribution cannot fit the error distribution of the models (represented by the histograms). This result has also been confirmed in multiple studies in the literature [28, 45]. Comparatively, the Laplace distribution can better fit the error distribution of the models as can be seen in Fig 10. Having a probability density model (like Laplace), that can fit the error distribution helps in generating a better and more accurate confidence interval, which is used to develop a new outlier detection method where the data inside the interval is labeled as normal data and data outside the interval is labeled as outliers. This outlier detection method is applied on DS1, where a 97% confidence interval is created using the Laplace distribution which is fitted to the error distribution of GBR as shown in Fig 11. Out of 47940 data samples, 993 data samples are labeled as outliers and are removed.

The value 97 is chosen after experimentation, because the more popular choice in literature [28, 52] e.g. 95% confidence interval tend to remove the normal data points as well, which results in the decrease in accuracy during the model training. Based on this outlier detection method, most of the hidden outliers that remained after pre-processing are removed.

## Conclusion

The reliability and accuracy of WTPC modeling results are affected by the presence of outliers in the training data. In this paper, error distributions of the WTPC model are studied, and it is found that the error distribution of a power curve is asymmetric, which indicates the presence of outliers. In this paper, a pre-processing technique is developed to remove the visible outliers; however, the error distribution of the modeled power curve remains asymmetrical because of the presence of hidden outliers that cannot be pre-processed. In this study, a novel WTPC modeling strategy is proposed to mitigate the adverse effect of the hidden outliers on WTPC modeling by developing multivariate power curve models. Multivariate models help in mitigating the adverse effect of hidden outliers because they incorporate information from additional inputs resulting in power curves that give a better representation of the dataset, hence achieving better accuracy in terms of MAE and RMSE. This is proved by analyzing the error distribution of the multivariate models compared with univariate models, which shows that the error distribution of multivariate models has higher peaks and lesser Standard deviation which indicates their errors are more converged to zero and have a lesser number of extreme outliers compared to the univariate case, hence eliminating the effect of hidden outliers.

In addition to solving the problem of hidden outliers, in this paper, Multivariate adaptive regression spline (MARS) is proposed for WTPC modeling. From the experimental results, it is concluded that:

- Non-parametric techniques perform much better compared with parametric techniques in terms of error metrics, such as MAE and RMSE, because of their data-driven nature.
- MARS outperforms all other benchmark techniques including the regression trees and achieves the best overall average rank (including all cases) in terms of accuracy. Also, it ranks above other techniques in both univariate and multivariate cases, hence it is concluded that MARS is the best WTPC modeling technique among others used in this study (in terms of accuracy). However, it is computationally more expensive than other techniques.
- Regression trees does not perform very well for univariate case. However, their performance improves for multivariate case in terms of their average ranking based on accuracy. Hence they can be a good choice for modeling multivariate power curves where lesser computational complexity is required, especially RFR and GBR (achieving second and third overall average rank).

Finally, it is proved the Laplace distribution is a better fit for the error distribution than the Gaussian distribution. Due to the superior fitting ability of the Laplace distribution, it is used to develop an outlier detection method to detect hidden outliers, by building a 97% confidence interval where the data samples outside the interval are labeled as outliers. This method is applied on the power curve of GBR for DS1 and 993 out of 47940 data samples are successfully labeled as outliers and are removed.

In the future, the proposed multivariate strategy can be studied by incorporating more input variables (by obtaining the dataset with multiple data features) and selecting the best inputs which contribute most to the accuracy by analyzing the correlation between them.

## References

[1] Belu R, Koracin D. Wind characteristics and wind energy potential in western Nevada. Renewable energy. 2009 Oct 1;34(10):2246–51.

[2] Shokrzadeh S, Jozani MJ, Bibeau E. Wind turbine power curve modeling using advanced parametric and nonparametric methods. IEEE Transactions on Sustainable Energy. 2014 Sep 8;5(4):1262–9.

[3] Council GW. Global wind report 2021. Global Wind Energy Council: Brussels, Belgium. 2021 Mar 24:6–7.

[4] Seo S, Oh SD, Kwak HY. Wind turbine power curve modeling using maximum likelihood estimation method. Renewable energy. 2019 Jun 1;136:1164–9.

[5] Zhao Y, Ye L, Wang W, Sun H, Ju Y, Tang Y. Data-driven correction approach to refine power curve of wind farm under wind curtailment. IEEE Transactions on Sustainable Energy. 2017 Jun 19;9(1):95–105.

[6] Rogers TJ, Gardner P, Dervilis N, Worden K, Maguire AE, Papatheou E, et al. Probabilistic modelling of wind turbine power curves with application of heteroscedastic Gaussian process regression. Renewable Energy. 2020 Apr 1;148:1124–36.

[7] Yan J, Zhang H, Liu Y, Han S, Li L. Uncertainty estimation for wind energy conversion by probabilistic wind turbine power curve modelling. Applied energy. 2019 Apr 1;239:1356–70.

[8] Hanoon MS, Ahmed AN, Kumar P, Razzaq A, Zaini NA, Huang YF, et al. Wind speed prediction over Malaysia using various machine learning models: potential renewable energy source. Engineering Applications of Computational Fluid Mechanics. 2022 Dec 31;16(1):1673–89.

[9] He Q, Wang J, Lu H. A hybrid system for short-term wind speed forecasting. Applied energy. 2018 Sep 15;226:756–71.

[10] Mi XW, Liu H, Li YF. Wind speed forecasting method using wavelet, extreme learning machine and outlier correction algorithm. Energy Conversion and Management. 2017 Nov 1;151:709–22.

[11] Wang Y, Hu Q, Li L, Foley AM, Srinivasan D. Approaches to wind power curve modeling: A review and discussion. Renewable and Sustainable Energy Reviews. 2019 Dec 1;116:109422.

[12] Goretti G, Duffy A, Lie TT. The impact of power curve estimation on commercial wind power forecasts—An empirical analysis. In2017 14th International Conference on the European Energy Market (EEM) 2017 Jun 6 (pp. 1–4). IEEE.

[13] Ezzat AA. Turbine-specific short-term wind speed forecasting considering within-farm wind field dependencies and fluctuations. Applied Energy. 2020 Jul 1;269:115034.

[14] Kusiak A, Zheng H, Song Z. On-line monitoring of power curves. Renewable Energy. 2009 Jun 1;34(6):1487–93.

[15] Carrillo C, Montaño AO, Cidrás J, Díaz-Dorado E. Review of power curve modelling for wind turbines. Renewable and Sustainable Energy Reviews. 2013 May 1;21:572–81.

[16] Lydia M, Kumar SS, Selvakumar AI, Kumar GE. A comprehensive review on wind turbine power curve modeling techniques. Renewable and Sustainable Energy Reviews. 2014 Feb 1;30:452–60.

[17] Taslimi-Renani E, Modiri-Delshad M, Elias MF, Rahim NA. Development of an enhanced parametric model for wind turbine power curve. Applied energy. 2016 Sep 1;177:544–52.

[18] Wang Y, Hu Q, Srinivasan D, Wang Z. Wind power curve modeling and wind power forecasting with inconsistent data. IEEE Transactions on Sustainable Energy. 2018 Mar 28;10(1):16–25.

[19] Marčiukaitis M, Žutautaitė I, Martišauskas L, Jokšas B, Gecevičius G, Sfetsos A. Non-linear regression model for wind turbine power curve. Renewable Energy. 2017 Dec 1;113:732–41.

[20] Trivellato F, Battisti L, Miori G. The ideal power curve of small wind turbines from field data. Journal of Wind Engineering and Industrial Aerodynamics. 2012 Aug 1;107:263–73.

[21] Gottschall J, Peinke J. How to improve the estimation of power curves for wind turbines. Environmental Research Letters. 2008 Jan 11;3(1):015005.

[22] Villanueva D, Feijóo A. Normal-based model for true power curves of wind turbines. IEEE Transactions on Sustainable Energy. 2016 Jan 26;7(3):1005–11.

[23] Feijóo A, Villanueva D. Four-parameter models for wind farm power curves and power probability density functions. IEEE Transactions on Sustainable Energy. 2017 Apr 27;8(4):1783–4.

[24] Pelletier F, Masson C, Tahan A. Wind turbine power curve modelling using artificial neural network. Renewable Energy. 2016 Apr 1;89:207–14.

[25] Ciulla G, D’Amico A, Di Dio V, Brano VL. Modelling and analysis of real-world wind turbine power curves: Assessing deviations from nominal curve by neural networks. Renewable energy. 2019 Sep 1;140:477–92.

[26] Schlechtingen M, Santos IF, Achiche S. Using data-mining approaches for wind turbine power curve monitoring: A comparative study. IEEE Transactions on Sustainable Energy. 2013 Feb 14;4(3):671–9.

[27] Ouyang T, Kusiak A, He Y. Modeling wind-turbine power curve: A data partitioning and mining approach. Renewable Energy. 2017 Mar 1;102:1–8.

[28] Wang Y, Hu Q, Pei S. Wind power curve modeling with asymmetric error distribution. IEEE Transactions on Sustainable Energy. 2019 Jun 3;11(3):1199–209.

[29] Kusiak A, Zheng H, Song Z. Models for monitoring wind farm power. Renewable Energy. 2009 Mar 1;34(3):583–90.

[30] Mehrjoo M, Jozani MJ, Pawlak M. Toward hybrid approaches for wind turbine power curve modeling with balanced loss functions and local weighting schemes. Energy. 2021 Mar 1;218:119478.

[31] Astolfi D, Castellani F, Lombardi A, Terzi L. Multivariate SCADA data analysis methods for real-world wind turbine power curve monitoring. Energies. 2021 Feb 19;14(4):1105.

[32] Guo P, Infield D. Wind turbine power curve modeling and monitoring with Gaussian process and SPRT. IEEE Transactions on Sustainable Energy. 2018 Dec 2;11(1):107–15.

[33] Janssens O, Noppe N, Devriendt C, Van de Walle R, Van Hoecke S. Data-driven multivariate power curve modeling of offshore wind turbines. Engineering Applications of Artificial Intelligence. 2016 Oct 1;55:331–8.

[34] Zou R, Yang J, Wang Y, Liu F, Essaaidi M, Srinivasan D. Wind turbine power curve modeling using an asymmetric error characteristic-based loss function and a hybrid intelligent optimizer. Applied Energy. 2021 Dec 15;304:117707.

[35] Lydia M, Selvakumar AI, Kumar SS, Kumar GE. Advanced algorithms for wind turbine power curve modeling. IEEE Transactions on sustainable energy. 2013 Apr 1;4(3):827–35.

[36] Manobel B, Sehnke F, Lazzús JA, Salfate I, Felder M, Montecinos S. Wind turbine power curve modeling based on Gaussian processes and artificial neural networks. Renewable Energy. 2018 Sep 1;125:1015–20.

[37] Yu F, Xu X. A short-term load forecasting model of natural gas based on optimized genetic algorithm and improved BP neural network. Applied Energy. 2014 Dec 1;134:102–13.

[38] Veena R, Mathew S, Petra MI. Artificially intelligent models for the site-specific performance of wind turbines. International Journal of Energy and Environmental Engineering. 2020 Sep;11:289–97.

[39] Bulaevskaya V, Wharton S, Clifton A, Qualley G, Miller WO. Wind power curve modeling in complex terrain using statistical models. Journal of Renewable and Sustainable Energy. 2015 Jan 1;7(1).

[40] Ghimire S, Alizadeh SM. Developing a decision tree algorithm for wind power plants siting and sizing in distribution networks. Energies. 2021 Apr 19;14(8):2293.

[41] Breiman L. Bagging predictors. Machine learning. 1996 Aug;24:123–40.

[42] Geurts P, Ernst D, Wehenkel L. Extremely randomized trees. Machine learning. 2006 Apr;63:3–42.

[43] Singh U, Rizwan M, Alaraj M, Alsaidan I. A machine learning-based gradient boosting regression approach for wind power production forecasting: A step towards smart grid environments. Energies. 2021 Aug 23;14(16):5196.

[44] Liu Z, Gao W, Wan YH, Muljadi E. Wind power plant prediction by using neural networks. In 2012 IEEE energy conversion congress and exposition (ECCE) 2012 Sep 15 (pp. 3154–3160). IEEE.

[45] Sun YZ, Wu J, Li GJ, He J. Dynamic economic dispatch considering wind power penetration based on wind speed forecasting and stochastic programming. Proceedings of the CSEE. 2009 Mar;29(4):41–7.

[46] Osman AI, Ahmed AN, Huang YF, Kumar P, Birima AH, Sherif M, et al. Past, present and perspective methodology for groundwater modeling-based machine learning approaches. Archives of Computational Methods in Engineering. 2022 Oct;29(6):3843–59.

[47] Hecht-Nielsen R. Kolmogorov’s mapping neural network existence theorem. In Proceedings of the international conference on Neural Networks 1987 Jun 21 (Vol. 3, pp. 11–14). New York, NY, USA: IEEE press.

[48] Alkesaiberi A, Harrou F, Sun Y. Efficient wind power prediction using machine learning methods: A comparative study. Energies. 2022 Mar 23;15(7):2327.

[49] Wan C, Cui W, Song Y. Probabilistic forecasting for power systems with renewable energy sources: Basic concepts and mathematical principles. Proceedings of the CSEE. 2021;41(19):6493–509.

[50] Irwan D, Ali M, Ahmed AN, Jacky G, Nurhakim A, Ping Han MC, et al. Predicting Water Quality with Artificial Intelligence: A Review of Methods and Applications. Archives of Computational Methods in Engineering. 2023 Jun 13:1–20.

[51] Ehteram M, Ahmed AN, Khozani ZS, El-Shafie A. Graph convolutional network–Long short term memory neural network-multi layer perceptron-Gaussian progress regression model: A new deep learning model for predicting ozone concertation. Atmospheric Pollution Research. 2023 Jun 1;14(6):101766.

[52] Hodge BM, Milligan M. Wind power forecasting error distributions over multiple timescales. In2011 IEEE power and energy society general meeting 2011 Jul 24 (pp. 1–8). IEEE.

[53] Kallner A. Laboratory statistics: methods in chemistry and health sciences. Elsevier; 2017 Oct 23.

[54] Siegel A. F. Variability: Dealing with Diversity. In Birtcher K. (Eds.). Practical business statistics (Eighth ed.). Academic Press. pp. 105–134.

[55] Marshall, H. Standard Deviation Formula and Uses vs. Variance. Investopedia. 2022. https://www.investopedia.com/terms/s/standarddeviation.

